# Early effects of ozoralizumab 30 mg in patients with rheumatoid arthritis and inadequate response to methotrexate: a post hoc trajectory analysis of the phase II/III OHZORA trial

**DOI:** 10.1136/rmdopen-2025-005710

**Published:** 2025-06-19

**Authors:** Yusuke Miyazaki, Nobuko Horiuchi, Shunsuke Okamoto, Rumiko Matsumoto, Tsutomu Takeuchi, Yoshiya Tanaka

**Affiliations:** 1 First Department of Internal Medicine, University of Occupational and Environmental Health, Japan, Kitakyushu, Japan; 2 Taisho Pharmaceutical Co Ltd, Tokyo, Japan; 3 Saitama Medical University, Iruma, Japan; 4 Keio University School of Medicine, Tokyo, Japan; 5 Department of Molecular Targeted Therapies, School of Medicine, University of Occupational and Environmental Health Japan, Kitakyushu, Fukuoka Prefecture, Japan

**Keywords:** Rheumatoid Arthritis, Tumor Necrosis Factor Inhibitors, Treatment

## Abstract

**Objective:**

This study assessed the early effects of ozoralizumab (OZR) 30 mg in patients with rheumatoid arthritis (RA) with inadequate response to methotrexate (MTX-IR), drawing on OHZORA trial data for efficacy and safety insights.

**Methods:**

The study included 141 patients with RA from the OHZORA trial, initiated on OZR 30 mg. The primary measure was the rate of achieving low disease activity (LDA) by the Clinical Disease Activity Index (CDAI) 3 days post-OZR initiation. Growth mixture modelling (GMM) of CDAI trajectories was performed to enable a more detailed analysis of the impact of OZR on disease activity improvement.

**Results:**

The retention rate of OZR up to 52 weeks was 87.9% (n=124). The LDA achievement rate on the third day of OZR introduction was 12.8% (n=18), and by week 52, 70.9% (n=100) had improved to LDA. Three distinct groups were identified using GMM: one group (n=78) reached LDA within 4 weeks of OZR initiation and maintained LDA up to week 52. Multiple logistic regression analysis revealed that both low baseline C-reactive protein (CRP) and low CDAI were independently associated with group membership.

**Conclusion:**

OZR 30 mg demonstrated both immediate and sustained efficacy in MTX-IR patients with RA. Multivariate analysis suggested that both baseline inflammation and disease activity—represented by CRP and CDAI—may independently influence treatment response. However, residual confounding due to baseline disease activity cannot be completely excluded, and this remains a limitation of the present study.

**WHAT IS ALREADY KNOWN ON THIS TOPIC**

- Treatment with ozoralizumab has been shown to rapidly improve disease activity in some patients with highly active rheumatoid arthritis (RA).

**WHAT THIS STUDY ADDS**

- By day 3 after ozoralizumab administration, 12.7% (18/141) of patients had already achieved low disease activity, demonstrating its rapid onset of action.
- This study also identified three distinct trajectories of disease activity in patients with RA treated with ozoralizumab using growth mixture modelling.
- The group with the highest efficacy had lower baseline C-reactive protein (CRP) levels and lower Clinical Disease Activity Index (CDAI) scores.

**HOW THIS STUDY MIGHT AFFECT RESEARCH, PRACTICE OR POLICY**

- This study highlights the potential of baseline CRP and CDAI as independent predictors of early and sustained response to ozoralizumab in patients with RA with inadequate response to methotrexate.
- These findings may help identify patients with RA who are more likely to benefit from ozoralizumab therapy, thereby supporting more individualised treatment decisions.

## Introduction

 Rheumatoid arthritis (RA) is a systemic inflammatory disease that causes progressive bone and joint destruction, leading to irreversible dysfunction, if left insufficiently treated.^1 2^ In the treatment of RA, the advent of biological disease-modifying antirheumatic drugs (bDMARDs) has caused a paradigm shift for the past 27 years since introduced in the USA. Tumour necrosis factor (TNF) inhibitors, which are the first type of bDMARDs approved for the treatment of RA, are still used as the first line of bDMARDs for patients with RA with an inadequate response to methotrexate (MTX-IR) because of their confirmed long-term efficacy and safety.^3^

Ozoralizumab (OZR) is the most recently approved TNF inhibitor for the treatment of patients with RA in Japan. OZR is also the first approved bispecific antibody with heavy-chain variable regions (VHH) and is called a NANOBODY compound. In OZR, two human TNF-α binding domains and one human serum albumin (HSA) binding domain are connected by a 9-amino-acid glycine-serine linker. OZR comprises variable domains and is characterised by the lack of Fc region.^4^ Although the HSA-binding domain contributes primarily to prolonging the serum half-life, its presence enables binding to two distinct targets, thereby meeting the criteria for bispecificity in VHH-based constructs. OZR with an HSA binding domain has possible advantages in the treatment of RA. The distribution of the drug to inflamed sites may be enhanced through binding to albumin accumulated in inflamed tissues.^5^ A study using a mouse model of arthritis reported that the distribution of mouse substitute antibodies for OZR to inflamed sites was enhanced through binding to albumin.^6^ Another study on fluorescence imaging of inflamed joints in a mouse model of collagen-induced arthritis reported that when fluorescence-labelled OZR was subcutaneously administered, the fluorescence intensity in the limbs was stronger in mice treated with OZR than that of mice treated with adalimumab. Furthermore, the amount of OZR distributed to inflamed sites was greater than that of adalimumab.^7^

Notably, the OHZORA trial^8^ showed that the disease activity on day 3 after the introduction of OZR was significantly lower in the OZR group than that in the placebo group. Based on the results of the OHZORA and NATSUZORA trials, OZR 30 mg was approved for the treatment of RA. However, the improvement achieved on the third day and the characteristics of patients who rapidly respond to OZR and maintain the improvement in disease activity are unknown. In patients with RA, early control of disease activity is essential for achieving long-term remission and favourable functional outcomes.^9 10^ Therefore, identifying the patients with RA and MTX-IR who respond to OZR is clinically meaningful.

Thus, this study aimed to perform a post hoc analysis of the OHZORA trial to determine the percentage of patients with RA and MTX-IR that achieved improvement in disease activity after the introduction of OZR 30 mg. We also analysed the characteristics of patients who maintained improvements in disease activity after a rapid response to treatment. Using the growth mixture modelling (GMM),^11^ an analytical method that identifies groups with different trajectories of factors changing over time, we determined the characteristics of a group that rapidly responded to OZR and maintained improvements in disease activity. Additionally, we evaluated the clinical characteristics of patients with RA and MTX-IR who responded to OZR.

## Material and methods

### Patients

The present study involved Japanese patients with RA aged 20 to 75 years who met the 2010 RA Classification Criteria of the American College of Rheumatology (ACR)^12^ and inadequately responded to MTX. The inclusion criteria were patients who had an active disease (defined as six joints or more on the 68-tender joint count [TJC68], six joints or more on the 66-swollen joint count [SJC66], 0.6 mg/dL or higher of high-sensitivity C-reactive protein (hs-CRP), or 28 mm/hour or higher of erythrocyte sedimentation rate (ESR)), had been treated with MTX for 12 weeks or longer before baseline, and had received MTX at the same dose (6–16 mg/week) for 6 weeks or longer before baseline. The exclusion criteria were as follows: patients with abnormal chest radiographic findings suggestive of malignant tumours, infection or interstitial pneumonia, patients with active tuberculosis, and patients with latent tuberculosis (defined as those with a previous history of tuberculosis or those confirmed with release of interferon γ, except those who had started antituberculosis drug therapy with isoniazid before the trial).

### Study design

The present study (JapicCTI-184029) was a multicentre randomised placebo-controlled double-blind parallel-group confirmatory study, consisting of a 24-week double-blind treatment period (period A) followed by a 28-week open-label treatment period (period B). It was conducted at 78 institutions in Japan from September 2018 to October 2020. Patients corresponding to the early escape criteria (less than 20% improvement in TJC68 and SJC66 from baseline) at week 16 of period A were transferred from the placebo group to the OZR 30 mg group (P/30 mg group) or from the OZR 30 mg group to the OZR 80 mg group (30/80 mg group) at week 20 under the double-blind condition of period B. Patients who received placebo for 24 weeks during period A were randomly reassigned at a ratio of 1:1 to receive OZR 30 mg (P/30 mg group) or OZR 80 mg (P/80 mg group). In this post hoc analysis, we included 141 patients who had been treated with OZR 30 mg and MTX from the start of period A in the OHZORA trial (n=152), excluding two patients with missing CDAI data from baseline to week 1, and nine patients who met early-escape criteria at week 20 and were switched to OZR 80 mg.

The present clinical study was conducted in compliance with the Declaration of Helsinki, the Law on Securing Quality, Efficacy and Safety of Products Including Pharmaceuticals and Medical Devices, and the ethical principles of clinical research.

### Clinical efficacy and outcome

The primary outcome was the rate of achievement of low disease activity (LDA) at day 3, measured by the Clinical Disease Activity Index (CDAI).^13 14^ CDAI remission was defined as a score of less than or equal to 2.8; LDA was defined as a score of less than or equal to 10.0. Additional secondary outcomes included disease activity, retention rate and safety at week 52.

### Safety

For safety assessment, adverse events (including injection site reactions, serious adverse events and adverse events to be noted) were evaluated.

### GMM

GMM^11^ was performed with STATA V.16.0 (StataCorp LLC, College Station, Texas, USA). Bayesian information criterion (BIC) was used to compare the goodness-of-fit of quadratic to quintic function-based linear models, and models with better goodness-of-fit were used. As for the number of groups, BIC was calculated for conditions in which there were two to seven groups, and the number of groups with the best goodness-of-fit was used. To include all patients in the analysis, the non-responder imputation method was used for patients who discontinued the medication before week 48.

### Other statistical analyses

Patients characteristics were expressed as mean±SD, median (IQR), or number (%) of patients. The Kaplan-Meier method was used to assess the retention rates, and the log-rank test analysed the differences. Student’s t-test, Mann-Whitney’s U test or Bonferroni method were used for between-group comparisons; the Fisher’s exact test was used for categorical variables. All reported p values are two-sided. The level of significance was p<0.05. All analyses were conducted using JMP V.15.0 (SAS Institute, Cary, North Carolina, USA) and SPSS software V.25.0 (SPSS, Chicago, Illinois, USA).

## Results

### Baseline demographics and clinical characteristics

Online supplemental table S1 shows the baseline characteristics of the 141 patients who participated in the OHZORA trial and received OZR 30 mg from the start of the trial through periods A and B. bDMARDs-naïve patients accounted for 73%, more than 80% of all patients were positive for rheumatoid factor and anti-cyclic citrullinated peptide (CCP) antibodies. All patients enrolled in the present study had active disease.

The retention rate of OZR was 87.9% (124/141) (online supplemental figure S1). Online supplemental table S2 shows events that led to the discontinuation of OZR. OZR was discontinued because of infection in three patients, malignant tumours in two patients (one with lung adenocarcinoma and one with acute T-cell leukaemia), and other adverse events in four patients.

### Efficacy OZR 30 mg

**Figure 1:**
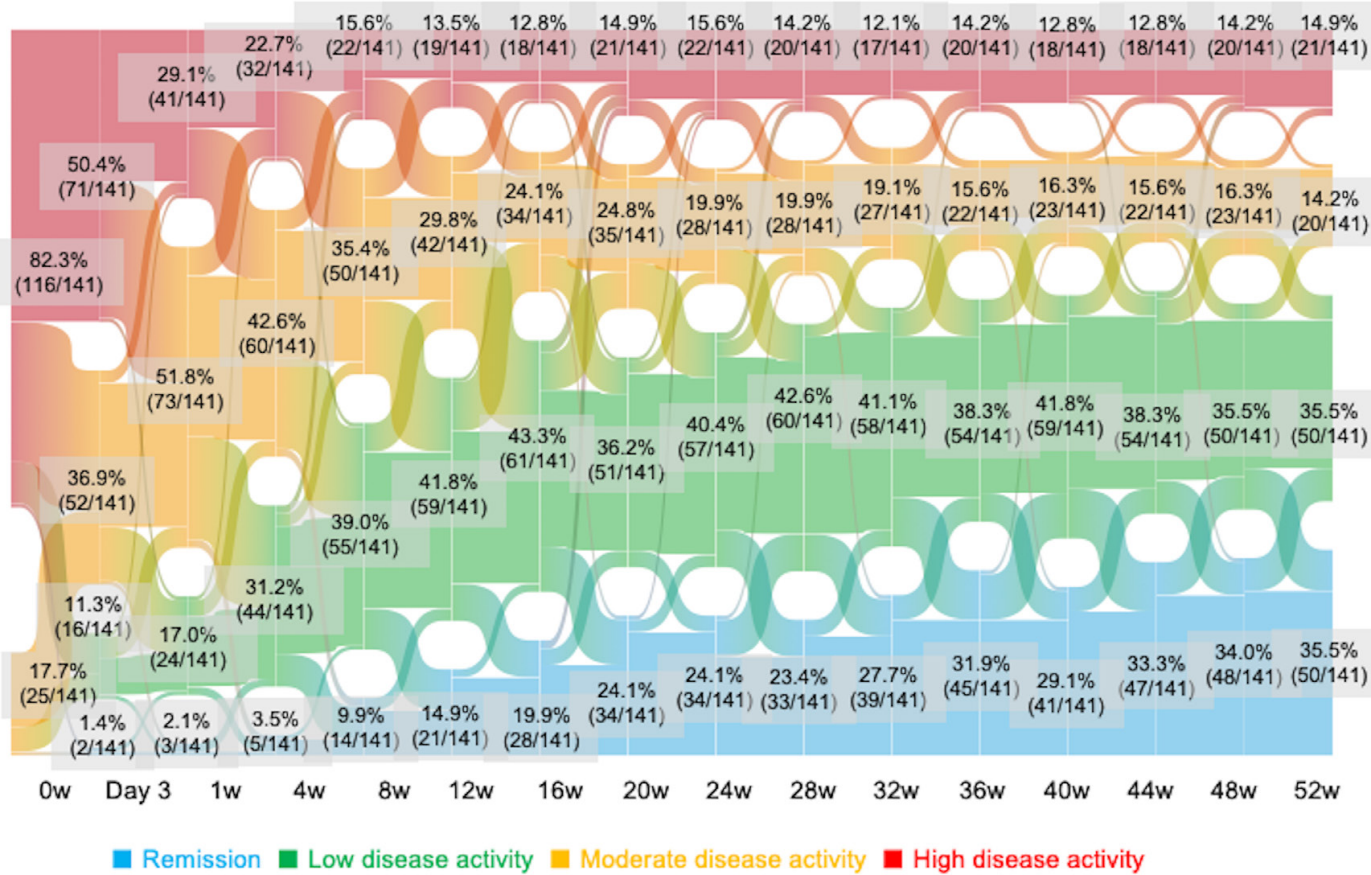
Changes in disease activity in patients with rheumatoid arthritis and inadequate response to methotrexate treated with ozoralizumab 30 mg in the OHZORA trial. Blue=remission; green=low disease activity; yellow=moderate disease activity; and red=high disease activity.

The changes in disease activity up to week 52 after the introduction of OZR are shown in a Sankey diagram (figure 1). Disease activity was high at baseline in 82.3% of the patients. After the introduction of OZR, disease activity improved to LDA or better in 18 patients with RA (12.7%) at day 3 and approximately 20% of all patients with RA at week 1. At week 52, remission was achieved in 35.5% of the patients, and disease activity improved to LDA or better in approximately 70.9% of the patients. To investigate factors associated with CDAI remission at week 52 across the entire study population, logistic regression analyses were conducted. In univariate analyses, lower baseline CDAI, HAQ-DI, CRP and ESR were associated with remission. In multivariate analysis adjusting for bDMARD-naïve status, baseline CDAI, CRP, rheumatoid factor, age and sex, both lower baseline CDAI (OR 0.91, 95% CI 0.86 to 0.96) and lower CRP (OR 0.74, 95% CI 0.52 to 0.99) remained independently associated with week 52 remission (online supplemental table S3).

### Trajectories of the CDAI in patients with RA treated with OZR 30 mg according to GMM

We analysed a subgroup of patients wherein OZR rapidly exerted therapeutic effects and controlled disease activity afterwards. To analyse the characteristics of this group, the trajectories of CDAI were analysed using GMM. This method is useful for identifying the characteristics of each trajectory group and analysing the factors affecting such trajectories.

**Figure 2:**
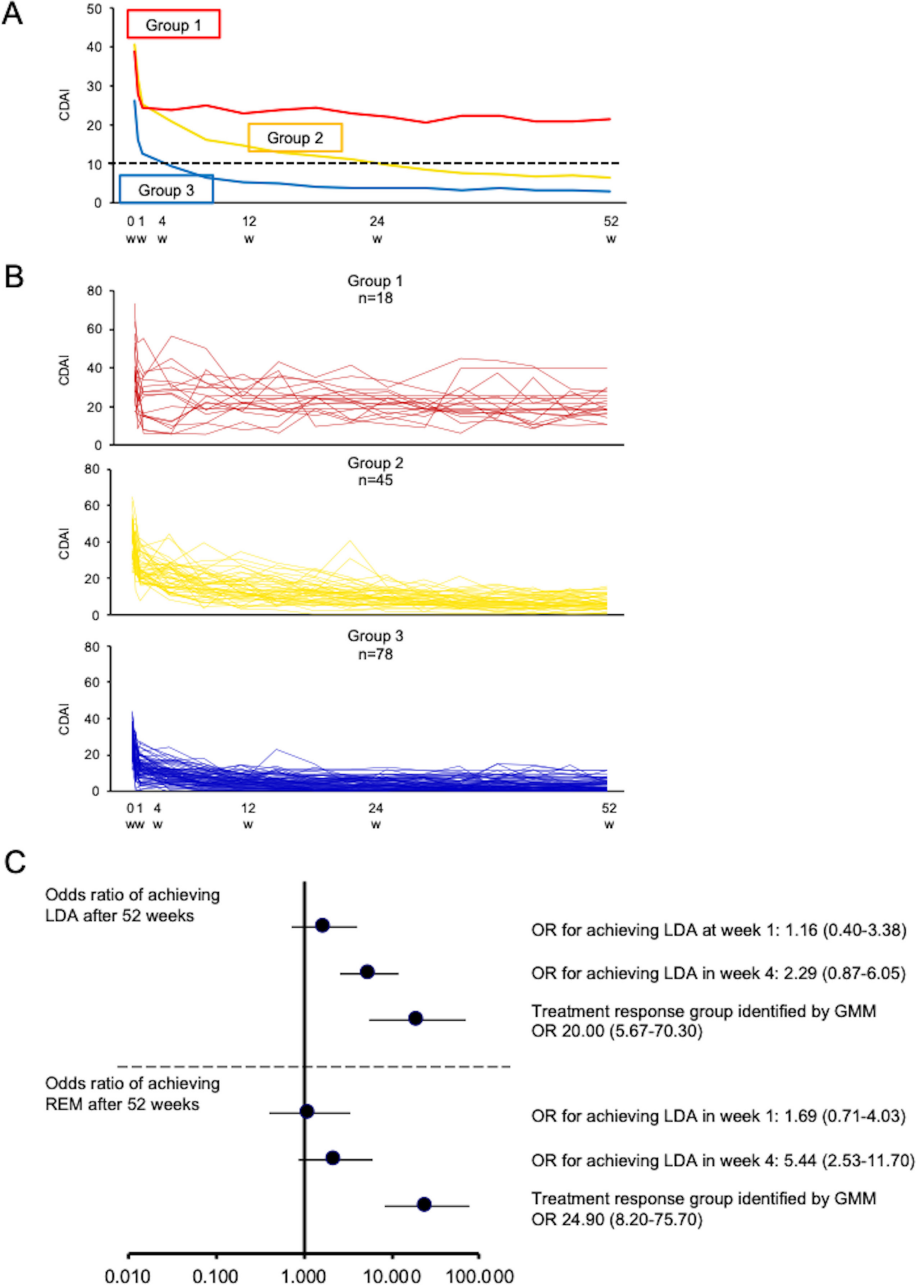
Growth mixture modelling (GMM) analysis of trajectories of the Clinical Disease Activity Index (CDAI) in patients with rheumatoid arthritis (RA) and inadequate response to methotrexate (MTX-IR) who were treated with ozoralizumab (OZR) 30 mg. (A) Changes in CDAI in the groups identified by GMM of trajectories of CDAI in patients with RA and MTX-IR who were treated with OZR 30 mg. Red line=group 1; yellow line=group 2; blue line=group 3. (B) Changes in CDAI in individual patients according to the groups identified by GMM. (C) ORs for achieving remission or low disease activity (LDA) at week 52 in the group of patients who achieved LDA at day 3 after the introduction of OZR, the group of patients who achieved LDA at week 1 after the introduction of OZR, and the treatment response group identified by GMM.

Using GMM, we analysed the trajectories of changes in CDAI in 141 patients with RA treated with OZR 30 mg. The best goodness-of-fit was observed for the cubic function-based linear model of the trajectory (online supplemental table S3). As for the number of groups, dividing the patients into three groups was associated with the best goodness-of-fit (online supplemental table S4). Thus, based on the results of GMM, the patients were divided into three trajectory groups (figure 2A). While the baseline disease activity was high in all groups, they were further divided into the groups with higher disease activity (groups 1; n=18 and 2; n=45) and the other group (group 3; n=78). In group 1, disease activity improved after the introduction of OZR, but LDA was not achieved at week 52. In group 2, LDA was achieved at week 24, and improvements in disease activity were maintained afterwards. In group 3, disease activity improved to LDA at week 4 and was maintained afterwards (the treatment response group) (figure 2B). Table 1 lists the baseline patient characteristics of the three trajectory groups identified by GMM. In the treatment response group, the values of TJC, SJC, patient’s global assessment of disease activity visual analogue scale (VAS), evaluator global assessment of disease activity (EGA), pain VAS, CDAI, health assessment questionnaire disability index (HAQ-DI), CRP, ESR, matrix metalloproteinase 3 (MMP-3) and serum interleukin-6 (IL-6) levels were low at the introduction of OZR.

**Table 1:** Characteristics of each trajectory group

Variables	Group 1 (poor response)n=18	Group 2 (late response)n=45	Group 3 (early response)n=78	P value
Age (years)	52.9±13.2	53.5±11.9	56.2±10.7	0.33
Sex, n (% female)	13 (72.2%)	30 (66.7%)	55 (70.5%)	0.87
Disease duration (month)	5.3±4.3	7.1±6.0	7.5±7.2	0.44
Treatment history				
MTX dose, mg/week	9.8±3.0	10.0±3.0	10.0±2.8	0.95
bDMARDs naïve, n (%)	15 (83.3%)	32 (71.1%)	56 (71.8%)	0.57
28-tender joint count	13.2±6.8	14.6±5.2	8.7±3.5	<0.0001
28-swollen joint count	11.7±7.1	12.6±5.1	8.2±2.7	<0.0001
GH, VAS 0–100 mm	75.7±19.8	67.1±17.2	52.5±19.8	<0.0001
EGA, VAS 0–100 mm	64.5±24.2	65.8±22.8	41.7±25.1	<0.0001
Pain VAS 0–100 mm	66.9±22.6	61.0±25.8	39.8±24.8	<0.0001
SDAI	41.6±15.6	42.7±11.3	27.3±7.3	<0.0001
CDAI	39.0±15.1	40.5±10.1	26.3±7.1	<0.0001
HAQ-DI	0.9±0.6	1.3±0.6	0.8±0.6	<0.0001
mTSS	21.9±20.1	30.3±33.9	22.0±32.0	0.35
CRP (mg/dL)	2.1 (0.6–4.0)	1.4 (0.4–3.1)	0.5 (0.2–1.3)	0.0007
ESR (mm/hour)	45.2±25.9	42.9±26.2	34.3±16.8	0.0393
Rheumatoid factor positive, n (%)	16 (88.9%)	38 (84.4%)	64 (82.1%)	0.76
Rheumatoid factor (U/mL)	44.0 (19.5–383.8)	57.0 (22.0–222.0)	43.0 (19.8–120.8)	0.65
Anti-CCP antibody, n (%)	17 (94.4%)	38 (84.4%)	72 (92.3%)	0.30
Anti-CCP antibody (U/mL)	291.0 (52.5–752.8)	158.0 (36.6–412.0)	84.2 (25.3–264.5)	0.16
MMP-3 (ng/mL)	159.1 (87.6–285.4)	143.1 (104.4–318.7)	131.2 (53.5–228.9)	0.0376
IL-6 (pg/mL)	40.4 (13.7–126.3)	45.3 (12.5–107.9)	8.5 (3.0–33.8)	<0.0001

Data are mean±SD, median (IQR) or number (%) of patients.

bDMARDs, biological disease-modifying anti-rheumatic drugs; CCP, cyclic citrullinated peptide; CDAI, Clinical Disease Activity Index; CRP, C-reactive protein; EGA VAS, Evaluator Global Assessment of Disease Activity Visual Analogue Scale; ESR, erythrocyte sedimentation rate; GH VAS, Patient’s Global Assessment of Disease Activity Visual Analogue Scale; HAQ-DI, Health Assessment Questionnaire Disability Index; IL-6, interleukin-6; MMP-3, matrix metalloproteinase 3; mTSS, Modified Total Sharp Score; MTX, methotrexate; SDAI, Simplified Disease Activity Index.

There was no significant difference in the proportion of bDMARD-experienced patients across the three trajectory groups (group 1: 3/18 (16.7%), group 2: 13/45 (28.9%), group 3: 22/78 (28.2%), p=0.57).

### Characteristics of the group identified by GMM wherein disease activity was rapidly improved by OZR

Among the groups divided according to the trajectories of CDAI identified by GMM, we focused on the treatment response group that showed rapid improvement in disease activity after the introduction of OZR 30 mg and achieved LDA at week 4. In this group, disease activity was improved to LDA or better in 96.2% of the patients (75/78) and remission was achieved in 62.8% (49/78) at week 52. To determine whether disease activity at week 52 could be predicted accurately in the treatment response group identified by GMM, we calculated ORs for achieving LDA or remission at week 52 in the groups that achieved LDA at week 1 or 4 after the introduction of OZR and the treatment response group (figure 2C). The OR for achieving LDA at week 52 was 20.00 (95% CI 5.67 to 70.30), and the OR for achieving remission was 24.90 (95% CI 8.20 to 75.70). These ratios indicated that improvements in disease activity at week 1 or 4 after the introduction of OZR, instead of achievement of LDA, could be predicted in the treatment response group identified by GMM. These findings further corroborate the reports by Smolen *et al*^15^ and Aletaha *et al*^16^ indicating that early improvements in disease activity are linked to better disease control at 1 year an association now also demonstrated in patients treated with OZR.

Multivariate analysis was performed to identify baseline factors associated with inclusion in the treatment response group (table 2). Univariate analyses identified CRP, ESR, IL-6, CDAI and factors directly associated with the calculation of CDAI. Then, multivariate analysis was performed with explanatory variables of bDMARD-naïveness, CDAI, Modified Total Sharp Score (mTSS), CRP, ESR, rheumatoid factor, MMP-3 and adjustment factors (sex and age), which appeared to be associated with the trajectories of CDAI. In addition, due to multicollinearity between CRP and IL-6 levels, only CRP, identified as a more significant factor for inclusion in the treatment response group by the bootstrap forest method, was selected. Multivariate analysis identified that lower CDAI (OR 0.85, 95% CI 0.80 to 0.90, p<0.0001)and lower CRP levels (OR 0.69, 95% CI 0.44 to 0.98, p=0.0389) were associated with inclusion in the treatment response group. When the CRP cut-off level for inclusion in the treatment response group was determined, patients with CRP levels of 1.35 mg/dL or lower were more likely to be included in this group (sensitivity: 0.78, specificity: 0.54, area under the curve: 0.69).

**Table 2:** Factors associated with inclusion in group 3

	Univariable analysis		Multivariable analysis	
	OR (95% CI)	P value	OR (95% CI)	P value
Age (years)	1.02 (0.99 to 1.05)	0.14	1.02 (0.98 to 1.06)	0.41
Sex (female)	1.11 (0.54 to 2.28)	0.77	0.57 (0.20 to 1.67)	0.30
Disease duration	1.02 (0.97 to 1.08)	0.40		
MTX dose, mg/week	1.01 (0.90 to 1.14)	0.84		
bDMARDs naïve	1.01 (0.52 to 2.15)	0.87	1.05 (0.542 to 2.64)	0.91
28-tender joint count	0.76 (0.69 to 0.85)	<0.0001		
28-swollen joint count	0.79 (0.71 to 0.87)	<0.0001		
GH, VAS 0–100 mm	0.95 (0.94 to 0.7)	<0.0001		
EGA, VAS 0–100 mm	0.96 (0.95 to 0.98)	<0.0001		
Pain VAS 0–100 mm	0.97 (0.95 to 0.98)	<0.0001		
CDAI	0.85 (0.80 to 0.90)	<0.0001	0.85 (0.80 to 0.90)	<0.0001
HAQ-DI	0.31 (0.17 to 0.56)	<0.0001		
mTSS	0.99 (0.98 to 1.01)	0.27	1.00 (0.98 to 1.01)	0.49
CRP (mg/dL)	0.64 (0.50 to 0.82)	<0.0001	0.69 (0.44 to 0.98)	0.0389
ESR (mm/hour)	0.98 (0.96 to 0.99)	0.0113	1.01 (0.99 to 1.05)	0.34
Rheumatoid factor (U/mL)	0.99 (0.99 to 0.99)	0.0219	1.00 (0.99 to 1.01)	0.87
Anti-CCP antibody (U/mL)	0.99 (0.99 to 1.01)	0.12		
MMP-3 (ng/mL)	0.99 (0.99 to 0.99)	0.0233	1.00 (0.99 to 1.01)	0.85
IL-6 (pg/mL)	0.99 (0.98 to 0.99)	<0.0001		

bDMARDs, biological disease-modifying anti-rheumatic drugs; CCP, cyclic citrullinated peptide; CDAI, Clinical Disease Activity Index; CRP, C-reactive protein; DAS, Disease Activity Score; EGA VAS, Evaluator Global Assessment of Disease Activity Visual Analogue Scale; EQ-5D, EuroQol five dimension; ESR, erythrocyte sedimentation rate; GH VAS, Patient’s Global Assessment of Disease Activity Visual Analogue Scale; HAQ-DI, health assessment questionnaire disability index; IL-6, interleukin-6; MMP-3, matrix metalloproteinase 3; mTSS, Modified Total Sharp Score; MTX, methotrexate; SDAI, Simplified Disease Activity Index.

## Discussion

The present study showed that the group of patients with RA and MTX-IR who were treated with OZR 30 mg included a subgroup that achieved LDA as early as day 3 of the treatment.

The rapid action of OZR has been attributed to its molecular structure, and the OHZORA trial confirmed its fast-acting clinical effects. In the present study, 18 patients (12.7%) achieved LDA at day 3 after the introduction of OZR 30 mg (online supplemental figure S2). The Rheumatoid Arthritis Prevention of Structural Damage (RAPID) 1 trial^17^ demonstrated that a significantly higher percentage of patients treated with certolizumab pegol, a fragment antibody similar to OZR, achieved a 20% improvement in ACR response criteria (ACR20) at week 1, compared with those treated with placebo. Furthermore, the Anti-TNF-Study Utilizing Biomarker Assay to Monitor Early Response to Certolizumab Pegol (TSUBAME) study,^18^ which was conducted by our research group, demonstrated that disease activity is significantly improved at day 1 after the introduction of certolizumab pegol. Although these studies suggest that fragment antibodies may generally exhibit fast-acting effects, only reports on OZR have shown that some patients with RA and MTX-IR achieved LDA at day 3 after treatment initiation in a placebo-controlled trial. Thus, OZR may be a very fast-acting drug due to its unique molecular structure.

Next, we used GMM to analyse the trajectories of CDAI and identify the characteristics of patients with RA and MTX-IR who were treated with OZR 30 mg whose disease activity improved rapidly and remained stable up to week 52. The results showed that 78 (55.3%) patients (group 3; treatment response group) achieved LDA at week four and maintained improvements in disease activity up to week 52, and they were found to have low CRP levels at baseline.

The present study has limitations. First, this study is a post-hoc analysis limited to patients who received 30 mg of OZR from the start of the trial, which introduces the potential for selection bias compared to the original randomized controlled trial design. Furthermore, as multiple additional analyses were conducted post-hoc without adjustment for multiplicity, all p-values should be interpreted as nominal and considered with caution. Second, the sample size is small. Given the limited sample sizes in the GMM-defined subgroups, the precision in identifying predictors of treatment response may be constrained. With a larger sample size, detailed characteristics of patients achieving LDA at day three after the introduction of OZR, as well as those who rapidly respond to OZR, could be identified. Third, the present study did not include a control group of patients treated with other TNF inhibitors. Although the study showed that OZR was more effective in patients with low CRP levels at treatment initiation, it is uncertain whether this is a feature specific to OZR or whether OZR has a faster onset of action in these patients compared to another anti-TNFi. Further analysis for other TNF inhibitors is needed to confirm this. Additionally, although this study demonstrated that OZR was capable of improving disease activity as early as Day 3 after initiation. However, previous trials on other TNF inhibitors typically assessed treatment responses starting from Week 2 or Week 4. Therefore, it remains unknown whether similar early effects could be observed with other TNF inhibitors at Day 3. A head-to-head study comparing OZR with other TNF inhibitors at Day 3 would be necessary to confirm whether its rapid onset of action is indeed superior. Fourth, in the group that achieved early and sustained LDA after OZR initiation, both baseline CRP and CDAI values were substantially lower. While multivariate analysis suggested these were independent predictors, it is difficult to completely rule out the contribution of lower baseline disease activity to the observed treatment response. Therefore, residual confounding due to baseline characteristics must be considered a limitation of this study. Finally, the MTX dose used in this study was lower than the average doses commonly used in Europe and the United States. In the C-OPERA trial,^19^ which evaluated the efficacy and safety of CZP plus MTX vs. placebo plus MTX in MTX-naïve Japanese patients with early RA, the optimal tolerable dose was approximately 12 mg/week. Additionally, studies using MTX-polyglutamate concentrations have shown that despite lower MTX doses, Japanese patients exhibit higher MTX-PG levels than Western patients.^20^ These findings suggest that the MTX dosage used in this study is clinically appropriate for Japanese patients.

In conclusion, OZR may have a rapid onset of action, with a subset of MTX-IR patients with RA achieving LDA as early as day 3. Our findings suggest that patients with lower baseline CRP or CDAI levels were more likely to respond rapidly to OZR and to sustain improvement in disease activity through week 52.

## Supplementary material

10.1136/rmdopen-2025-005710 online supplemental file 1

10.1136/rmdopen-2025-005710 online supplemental file 2

10.1136/rmdopen-2025-005710 online supplemental file 3

## Data Availability

Data are available upon reasonable request.
