# Supplementary material for: Early effects of ozoralizumab 30 mg in patients with rheumatoid arthritis and inadequate response to methotrexate: a post hoc trajectory analysis of the phase II/III OHZORA trial
Source: RMD Open. 2025 Jun 19;11(2):e005710. doi: 10.1136/rmdopen-2025-005710 (PMC12182106; doi:10.1136/rmdopen-2025-005710)
Supplement: online supplemental file 1 [file rmdopen-11-2-s001.docx]

**Supplementary Materials**

Supplementary Figure S1. Retention rate of ozoralizumab in patients with rheumatoid arthritis and inadequate response to methotrexate who were treated with ozoralizumab 30 mg in the OHZORA trial.

Supplementary Figure S2. Changes in clinical disease activity index in patients with rheumatoid arthritis and inadequate response to methotrexate treated with ozoralizumab 30 mg in the OHZORA trial, highlighting cases achieving low disease activity by Day 3 (red line).

Supplementary Table S1. Baseline characteristics of patients participating in the OHZORA trial.

| Variables | Patients treated with OZR 30 mg  (n=141) |
| --- | --- |
|  |  |
| Age (years) | 54.9 ± 11.5 |
| Sex, n (% female) | 98 (69.5%) |
| Disease duration (month) | 7.0 ± 6.5 |
| Treatment history |  |
| MTX dose, mg/w | 9.9 ± 3.0 |
| bDMARDs naïve, n (%) | 103 (73.0%) |
| 28-tender joint count | 11.1 ± 5.4 |
| 28-swollen joint count | 10.1 ± 4.9 |
| GH, VAS 0-100 mm | 50.1 ± 27.3 |
| EGA, VAS 0-100 mm | 52.4 ± 26.9 |
| Pain VAS 0-100 mm | 50.1 ± 27.3 |
| SDAI | 34.0 ± 12.5 |
| CDAI | 32.5 ± 11.6 |
| HAQ-DI | 1.0 ± 0.6 |
| mTSS | 10.8 (3.1-35.1) |
| CRP (mg/dL) | 0.8 (0.3-2.2) |
| ESR (mm/h) | 38.1 ± 21.5 |
| Rheumatoid factor positive, n (%) | 118 (83.7%) |
| Rheumatoid factor (U/mL) | 47.0 (20.3-149.3) |
| Anti-CCP antibody, n (%) | 126 (89.4%) |
| Anti-CCP antibody (U/mL) | 119.0 (31.4-390.8) |
| MMP-3 (ng/mL) | 140.9 (63.9–235.6) |
| IL-6 (pg/ml) | 17.6 (4.8-65.6) |

Data are mean ± SD, median (IQR), or number (%) of patients.

MTX: methotrexate, bDMARDs: biological disease modifying anti-rheumatic drugs, GH VAS: patient’s global assessment of disease activity visual analogue scale, EGA VAS: evaluator global assessment of disease activity visual analogue scale, DAS: disease activity score, SDAI: simplified disease activity index, CDAI: clinical disease activity index, HAQ-DI: health assessment questionnaire disability index, EQ-5D: EuroQol 5 dimension, CRP: C-reactive protein, ESR: erythrocyte sedimentation rate, CCP: cyclic citrullinated peptide, MMP-3: matrix metalloproteinase 3, IL-6: interleukin-6, mTSS: modified total sharp score.

Supplementary Table S2. Reasons for discontinuation of ozoralizumab.

| Variables | Patients treated with OZR 30 mg  n=141 |
| --- | --- |
| Patients discontinuing treatment, n (%) | 17 (12.1%) |
| Infection, n (%) | 3 (2.1%) |
| Malignancy, n (%) | 2 (1.4%) |
| Other adverse events, n (%) | 4 (2.8%) |
| Patient's request for discontinuation, n, (%) | 4 (2.8%) |
| Others, n (%) | 4 (2.8%) |

Data are the number (%) of patients.

Supplementary Table S3. Factors associated with remission at 52 weeks.

|  | univariable analysis | | multivariable analysis | |
| --- | --- | --- | --- | --- |
|  | Odds ratio (95% CI) | p value | Odds ratio (95% CI) | p value |
| Age (years) | 1.02 (0.99–1.05) | 0.26 | 1.01 (0.97-1.05) | 0.63 |
| Sex (female) | 0.67 (0.32–1.42) | 0.30 | 0.79 (0.10-1.50) | 0.23 |
| Disease duration | 1.00 (0.95–1.05) | 0.99 |  |  |
| MTX dose, mg/w | 1.06 (0.94–1.20) | 0.34 |  |  |
| bDMARDs naïve | 0.79 (0.37–1.72) | 0.87 | 0.67 (0.26-1.74) | 0.42 |
| 28-tender joint count | 0.84 (0.76–0.91) | <0.0001 |  |  |
| 28-swollen joint count | 0.88 (0.80–0.96) | 0.0037 |  |  |
| GH, VAS 0-100 mm | 0.98 (0.97–0.99) | 0.0477 |  |  |
| EGA, VAS 0-100 mm | 0.98 (0.96-0.99) | 0.0011 |  |  |
| Pain VAS 0-100 mm | 0.98 (0.97–0.99) | 0.0040 |  |  |
| CDAI | 0.92 (0.88–0.96) | <0.0001 | 0.91 (0.86-0.96) | <0.0001 |
| HAQ-DI | 0.51 (0.28–0.89) | 0.0177 |  |  |
| mTSS | 0.99 (0.98–1.01) | 0.27 |  |  |
| CRP (mg/dL) | 0.71 (0.53–0.90) | 0.0026 | 0.74 (0.52-0.99) | 0.0405 |
| ESR (mm/h) | 0.98 (0.96-0.99) | 0.0054 |  |  |
| Rheumatoid factor (U/mL) | 0.99 (0.99–1.01) | 0.42 | 1.00 (0.99-1.01) | 0.35 |
| Anti-CCP antibody (U/mL) | 0.99 (0.99–1.01) | 0.26 |  |  |
| MMP-3 (ng/mL) | 0.99 (0.99–1.01) | 0.08 |  |  |
| IL-6 (pg/ml) | 0.99 (0.98–1.01) | 0.08 |  |  |

MTX: methotrexate, bDMARDs: biological disease modifying anti-rheumatic drugs, GH VAS: patient’s global assessment of disease activity visual analogue scale, EGA VAS: evaluator global assessment of disease activity visual analogue scale, DAS: disease activity score, SDAI: simplified disease activity index, CDAI: clinical disease activity index, HAQ-DI: health assessment questionnaire disability index, EQ-5D: EuroQol 5 dimension, CRP: C-reactive protein, ESR: erythrocyte sedimentation rate, CCP: cyclic citrullinated peptide, MMP-3: matrix metalloproteinase 3, IL-6: interleukin-6, mTSS: modified total sharp score.

Supplementary Table S4. Determination of function of trajectory groups for CDAI.

|  | BIC | AIC |
| --- | --- | --- |
| Linear | 5480.32 | 5474.79 |
| Quadratic | 5448.03 | 5440.67 |
| Cubic | 5423.40 | 5412.35 |
| Quartic | 5453.60 | 5424.39 |
| Quintic | 5466.12 | 5433.32 |

AIC: Akaike information criterion; BIC: Bayesian information criterion.

Supplementary Table S5. Determination of the number of trajectory groups for CDAI.

| No. of groups | BIC | AIC |
| --- | --- | --- |
| 2 | 5242.89 | 5224.47 |
| 3 | 5135.12 | 5024.53 |
| 4 | 5189.47 | 5150.94 |
| 5 | 5188.25 | 5151.63 |
| 6 | 5182.78 | 5191.12 |
| 7 | 5184.46 | 5213.89 |

AIC: Akaike information criterion; BIC: Bayesian information criterion.

Supplementary Table S6. Disease activity at each time point by trajectory group

|  | Group 1 (poor response), n=18 | |
| --- | --- | --- |
|  | Remission, n (%) | Low disease activity, n (%) |
| Week 4 | 0 (0.0%) | 4 (22.2%) |
| Week 24 | 0 (0.0%) | 0 (0.0%) |
| Week 52w | 0 (0.0%) | 0 (0.0%) |
|  | Group 2 (late response), n =45 | |
|  | Remission, n (%) | Low disease activity, n (%) |
| Week 4 | 0 (0.0%) | 1 (2.2%) |
| Week 24 | 2 (4.4%) | 18 (40.0%) |
| Week 52w | 4 (8.9%) | 28 (62.2%) |
|  | Group 3 (early response), n =78 | |
|  | Remission, n (%) | Low disease activity, n (%) |
| Week 4 | 5 (6.4%) | 39 (50.0%) |
| Week 24 | 32 (41.0%) | 39 (50.0%) |
| Week 52w | 46 (59.0%) | 22 (28.2%) |
