# Supplementary figures and images for: Early effects of ozoralizumab 30 mg in patients with rheumatoid arthritis and inadequate response to methotrexate: a post hoc trajectory analysis of the phase II/III OHZORA trial
Source: RMD Open. 2025 Jun 19;11(2):e005710. doi: 10.1136/rmdopen-2025-005710 (PMC12182106; doi:10.1136/rmdopen-2025-005710)

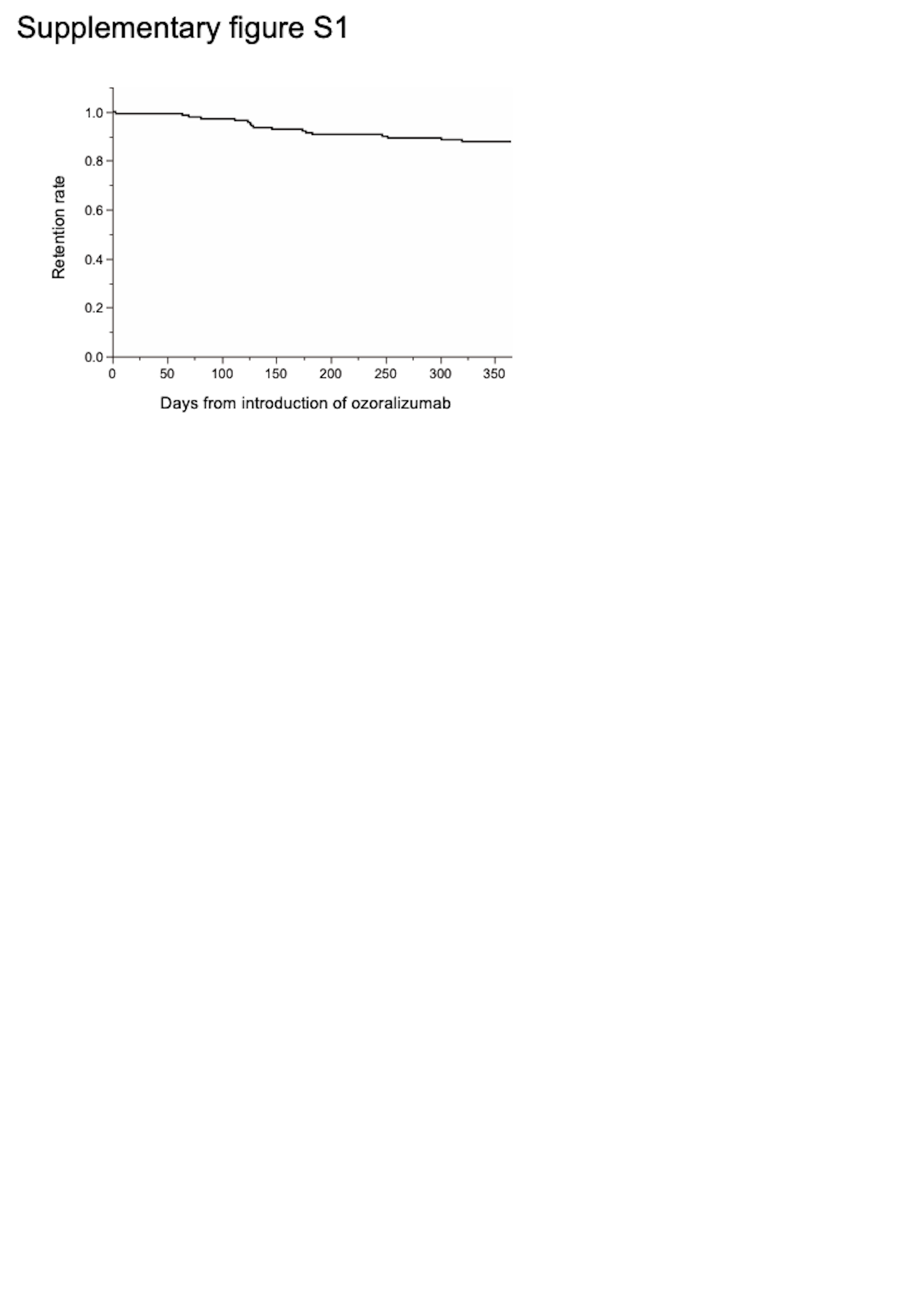

Supplement: online supplemental file 2 [file rmdopen-11-2-s002.tiff]

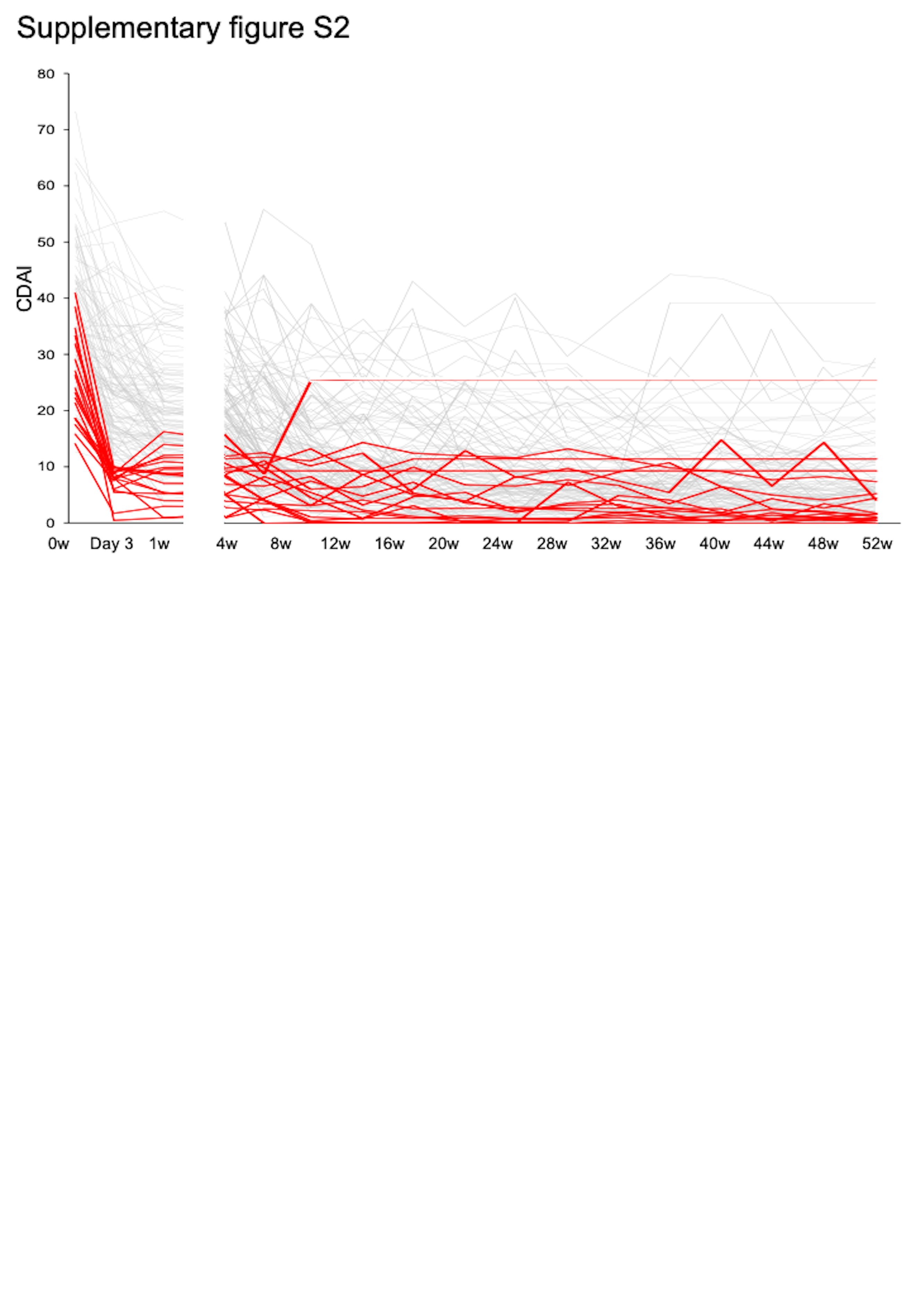

Supplement: online supplemental file 3 [file rmdopen-11-2-s003.tiff]
